# Computational study of the unimolecular and bimolecular decomposition mechanisms of propylamine

**DOI:** 10.1038/s41598-020-68723-7

**Published:** 2020-07-16

**Authors:** Mansour H. Almatarneh, Rima Al Omari, Reema A. Omeir, Ahmad Al Khawaldeh, Akef T. Afaneh, Mutasem Sinnokrot, Alaa Al Akhras, Ali Marashdeh

**Affiliations:** 1 Department of Chemistry, University of Jordan, Amman, 11942 Jordan; 2 Department of Chemistry, Memorial University, St. John’s, NL A1B 3X7 Canada; 3 Pharmacological and Diagnostic Research Centre (PDRC), Faculty of Pharmacy, Al-Ahliyya Amman University, Amman, 19328 Jordan; 4 Department of Chemistry, Faculty of Science, Al-Balqa’ Applied University, Salt, Jordan; 5 Department of Chemistry, Khalifa University-SAN Campus, 2533 Abu Dhabi, United Arab Emirates

**Keywords:** Physical chemistry, Theoretical chemistry

## Abstract

A detailed computational study of the dehydrogenation reaction of *trans*-propylamine (*trans*-PA) in the gas phase has been performed using density functional method (DFT) and CBS-QB3 calculations. Different mechanistic pathways were studied for the reaction of *n*-propylamine. Both thermodynamic functions and activation parameters were calculated for all investigated pathways. Most of the dehydrogenation reaction mechanisms occur in a concerted step transition state as an exothermic process. The mechanisms for pathways **A** and **B** comprise two key-steps: H_2_ eliminated from PA leading to the formation of allylamine that undergoes an unimolecular dissociation in the second step of the mechanism. Among these pathways, the formation of ethyl cyanide and H_2_ is the most significant one (pathway **B**), both kinetically and thermodynamically, with an energy barrier of 416 kJ mol^−1^. The individual mechanisms for the pathways from **C** to **N** involve the dehydrogenation reaction of PA via hydrogen ion, ammonia ion and methyl cation. The formation of α-propylamine cation and NH_3_ (pathway **E**) is the most favorable reaction with an activation barrier of 1 kJ mol^−1^. This pathway has the lowest activation energy calculated of all proposed pathways.

## Introduction

Propylamine is of significant importance in chemistry, as it constitutes a central structure block for aliphatic amines^1^. It is widely utilized as a solvent in organic synthesis, and as a finishing agent for drugs, rubber, fiber, paints, pesticides, textile and resin^2,3^, and in the generation of fungicides^4–6^. Furthermore, it may very well be used as a petroleum additive and preservative. The disintegration of protonated of propylamine has attracted a noteworthy arrangement of fascination in the previous decade^7–10^. This is mainly due to the way that the proton affinity and the structural difference in propylamine through protonation influences the separation items through the arrangement of protonated amines, methane, propene and hydrogen gas^11,12^. Additionally, this reaction prompts the generation of various poisonous synthetic substances such as, alkyl cyanide, propylene, ethylene, nitrogen and hydrogen gases^13,14^.

Protonation (B + H^+^  →  BH^+^) and deprotonation (dehydrogenation) (HA − H^+^  → A^−^) reactions assume a significant role in natural science and organic chemistry, where A and B are the acidic and the basic centers, respectively. They are considered as the first step in several fundamental chemical mechanisms elucidated in the cited reference^15^. The ability of an atom or molecule in the gas phase to accept or to lose a proton can be described by calculating the proton affinity (PA), deprotonation (dehydrogenation) enthalpy, and molecular gas-phase basicity, which offer a profound understanding of the connections between the reactivity of the organic molecules, their molecular structures, and molecular stability^16^. The negative of the enthalpy change related to the gas-phase protonation reaction is referred to as proton affinity, while dehydrogenation energy is defined as the enthalpy change related to the gas-phase dehydrogenation reaction^17^.

**Figure 1:**
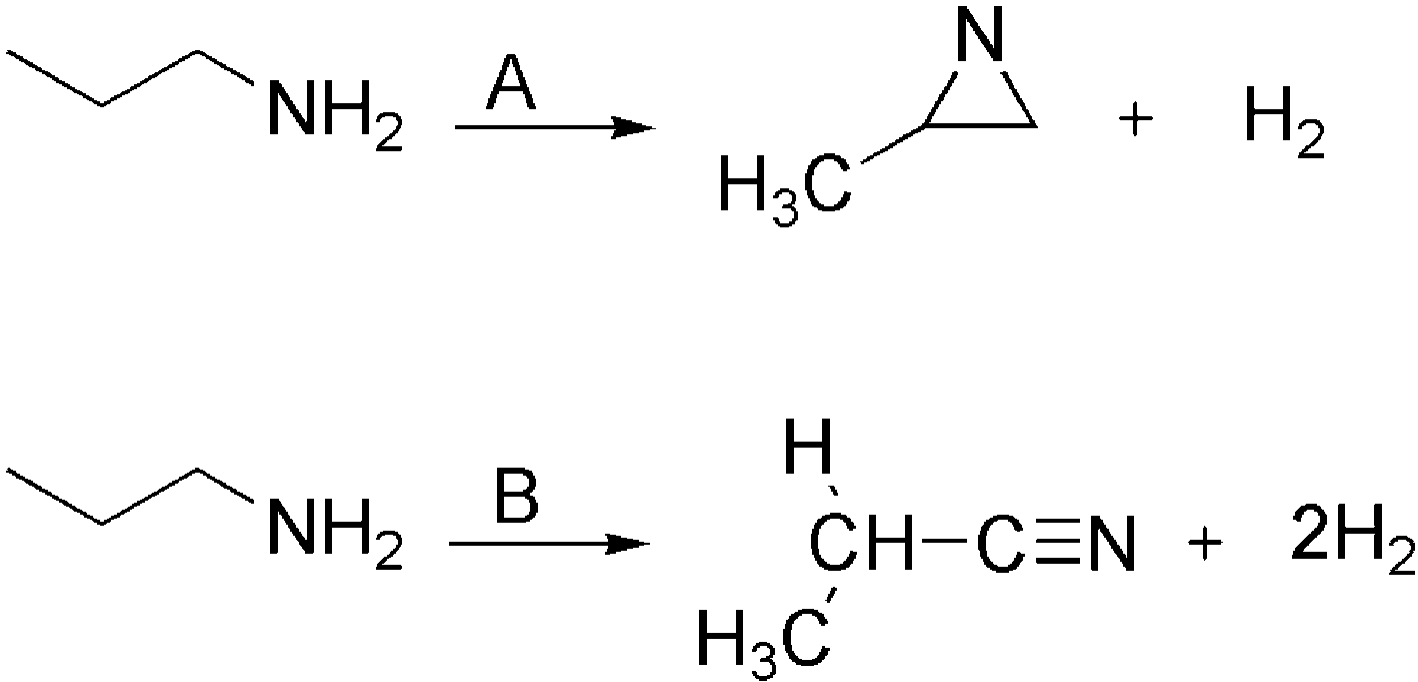
The proposed unimolecular dissociation of *n*-PA.

**Figure 2:**
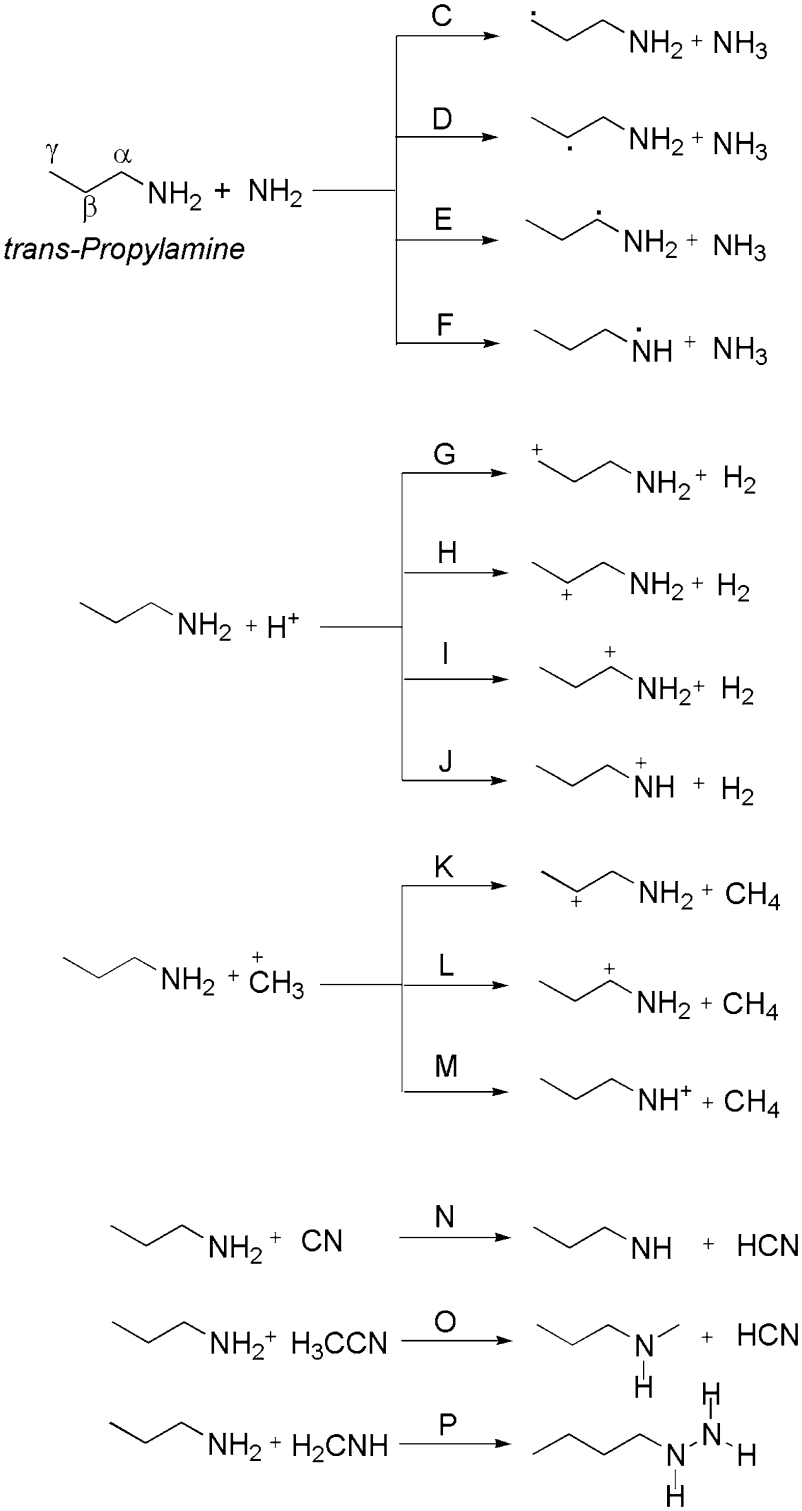
The proposed bimolecular reactions of *trans*-PA.

To the best of our knowledge, the dehydrogenation of *n*-propylamine has not been investigated or reported anywhere in the literature. Therefore, our main goal in this work is to calculate the relative stabilities of the possible tautomeric forms of PA in deprotonated cases in the gas-phase. Another goal is also to investigate the isomerization process resulting from the most stable species to the other tautomeric entities. Figures 1 and 2 depict the studied proton transfer reactions for propylamine.

## Computational methods

Geometry optimizations were performed using the Gaussian-16 (G16) quantum chemistry package^18^. All the reactants (R), transition states (TS), intermediates (I), and products (P) were fully optimized with no constraints. In order to confirm that the resulting geometries correspond to minima or TS’s on the potential energy surfaces, vibrational frequencies were computed. Therefore, TS’s were confirmed with one imaginary frequency. Geometry optimizations were performed using Becke’s three-parameter hybrid method using the LYP correlation functional (denoted B3LYP)^19,20^. Double-zeta and triple-zeta basis sets were used, namely, 6-31G(d), and 6-311G++(3df,3pd)^21^. Thereafter, single-point energy calculations were performed using CBS-QB3^22^. These levels of theory based on our previous studies of the decomposition pathways of different amines^23–26^. Relative energies of all stationary points were revised with zero-point vibrational energies (ZPE). Furthermore, transition states were evaluated using the intrinsic reaction coordinate (IRC) method using the B3LYP/6-31G(d) to connect a given transition state structures to local minima of the reactants and products/intermediates on the potential energy diagrams (PED’s)^27^. The potential energy diagram analysis aims to locate stationary structures of reaction mechanisms. To the extent that the whole species in the reaction pathways in this paper are volatile organic compounds, the geometry optimizations, frequencies, and IRCs for the gas-phase reactions were carried out at mentioned levels of theory.

## Results and discussion

In this study, extensive quantum chemistry calculations for sixteen reaction pathways are proposed for gas-phase proton transfer pathways of propylamine. Pathways **A** and **B** include unimolecular dissociation of PA (Fig. 1). Whiles pathways **C **→ **N** comprise the dehydrogenation reaction of PA with H^+^, ^−^NH_2_, ^+^CH_3,_ and CN^−^ as depicted in Fig. 2. In addition, the bimolecular reactions of PA with acetonitrile and methanimine have been investigated and are denoted as pathways **O** and **P** (Fig. 2). The kinetic parameters [activation energies (E_a_) and Gibbs energies of activation (ΔG^‡^)] for the studied pathways were calculated at various levels of theory (Tables 1 → 5). The most plausible pathways were determined using the calculated kinetic energies; the ones with lower values are considered the most favorable. The stationary points are plotted on a potential energy diagram for related pathways to characterize the energies of the most favorable reactions. On the PED, the gradients of these structures are zero along all of the internal coordinates. The PED characterizes the energy of a molecular assembly and its value depends on the coordinates of all the atoms in the molecular system. We should point out that the energy values on the PED (E_rel_) are the internal energy with respect to the reactant. It will be noted that α-, β-, and γ-carbons of propylamine with respect to the nitrogen atom are shown in certain pathways.

### Unimolecular dissociation of n-PA

**Figure 3:**
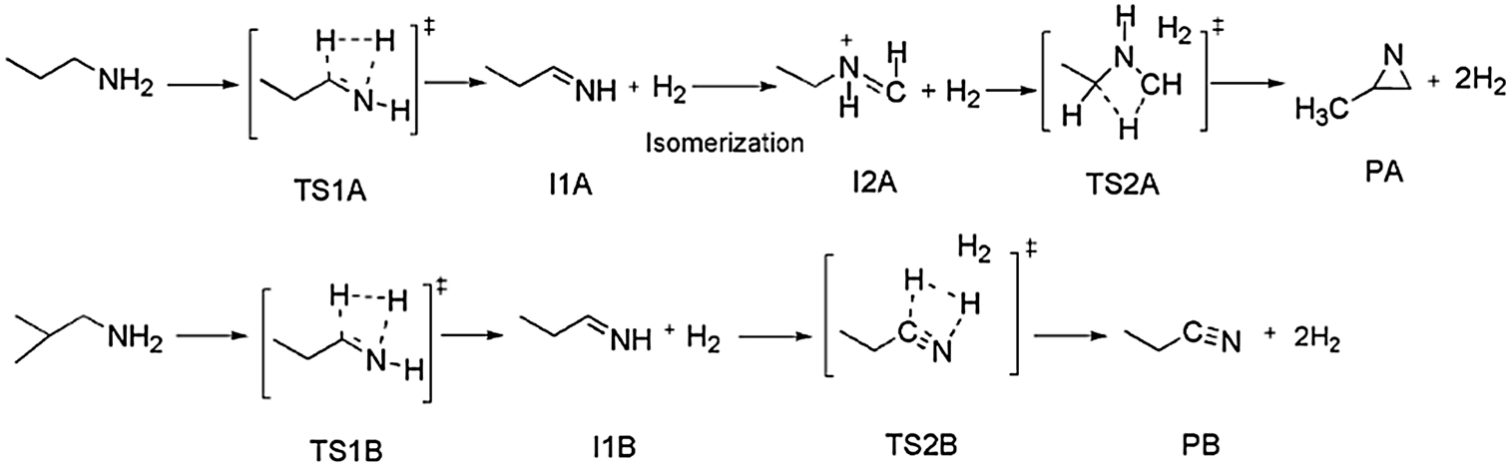
Unimolecular dissociation mechanism of PA for pathways **A** and **B**.

There are two possible pathways for the unimolecular dissociation reaction of PA which have been outlined as pathways **A** and **B **(Fig. 3). The pathway **A** involves a two-step mechanism. The first step is a formation of an intermediate I1 as an initial pre-reactive complex through TS1. In the first transition state, H_2_ is eliminated from the terminal nitrogen atom of PA and from α-carbon to form an unsaturated bond. As shown in Fig. 3, I1A undergoes isomerization to form I2A where both intermediates have the same number of atoms. The I1A and I2A intermediates are considered tautomers. These intermediates have different connectivity and arrangement of atoms, where the nitrogen forms a new bond with the α-carbon and simultaneously a broken bond between the α- and β-carbons. In the second step of the mechanism, three-membered-ring transition state (TS2A) is dominant, resulting in the formation of 2-methylaziridine, as shown in pathway **A** in Fig. 3.

In chemistry isomerization is the process by which one molecule is transformed into another molecule which has exactly the same atoms, these intermediates have a different arrangement of atoms.

The elimination of two H_2_ molecules and propionitrile formation are denoted in pathway **B**, Fig. 2. As the first step of the reaction is similar to pathway **A**, a tautomerization does not occur in an intermediate formation step. Therefore, another H_2_ molecule will be eliminated from I1B via TS2B, resulting in the formation of propionitrile as a product (Fig. 2).

**Figure 4:**
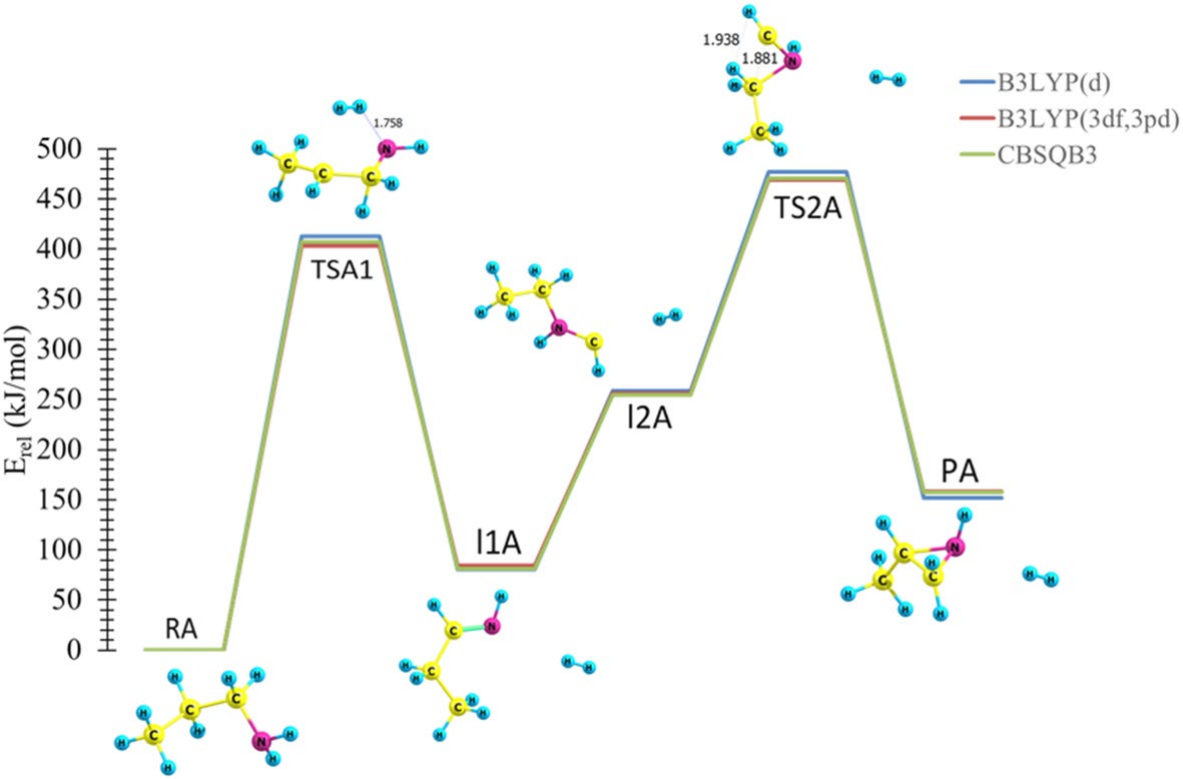
The PED for the dissociation reaction of Propylamine (pathway **A**). Energies calculated at different levels of theory. Optimized structures at B3LYP/6-311++G(3df,3dp).

**Figure 5:**
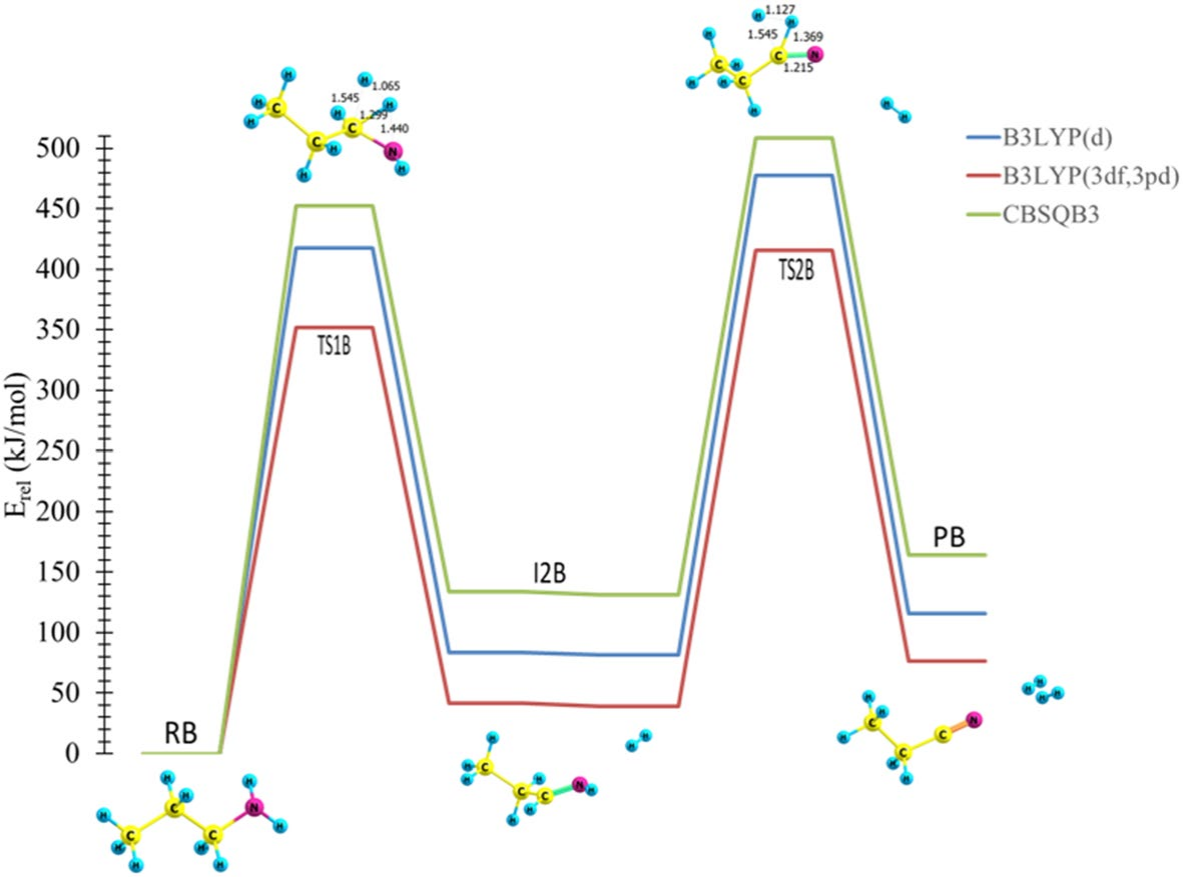
The PED for the dissociation reaction of Propylamine (pathway **B**). Energies calculated at different levels of theory. Optimized structures at B3LYP/6-311++G(3df,3dp).

In TS1A, a noticeable geometric change can be detected. For instance, the C–H and N–H bonds are elongated by about 0.904 Å. On the other hand, the H atoms approach each other, and the distance between them is shown to decrease by about 0.814 Å. The double bond has been formed with length of 1.375 Å. TS2A shows how the C–C and C–H bond lengths decreased to 1.881 Å and 1.938 Å, respectively. TS2B indicates that the C–H and N–H bond lengths increase to 1.545 Å and 1.369 Å, respectively, while the H–H bond length decreases to 1.127 Å. A triple bond is formed with length of 1.215 Å. The optimized structures for reactions coordinate (Pathways **A** and **B**) are plotted on potential energy diagrams at different levels of theory, and the reader is referred to Figs. 4 and 5.

In the first step for both pathways, the calculated activation energies are 407 and 403 kJ mol^−1^, at B3LYP/6-311G++(3df,3pd) and CBS-QB3 levels of theory, respectively, see Table 1. The respective activation energies of the rate-determining step TS2A and TS2B at the CBS-QB3 are 469 and 416 kJ mol^−1^, respectively. Furthermore, the respective activation energies at the B3LYP/6-31G(d) level of theory are 477 and 478 kJ mol^−1^ for TS2A and TS2B, respectively**.**

**Table 1:** Kinetic parameters (E_a_ and ΔG^‡^) for the unimolecular dissociation of *trans*-PA (in kJ mol^−1^) at 298.15 K.

Transition States	B3LYP/6-31G(d)		B3LYP/6-311++G(3df,3pd)		CBS-QB3	
	Ea	ΔG^‡^	Ea	ΔG^‡^	Ea	ΔG^‡^
TS1	413	416	407	408	403	404
TS2A	477	463	471	457	469	451
TS2B	478	457	509	489	416	390

It is worth noting that all bond lengths in TS1, TS2A, and TS2B are in excellent agreement with the reported studies for the unimolecular dissociation reactions of propylamine and protonated propylamine^28^. The thermodynamic properties of these pathways were found to be endothermic and endergonic with all levels employed. This indicates that the reaction is favorable in the reverse direction.

### Bimolecular dissociation of *n*-PA (protonation of *n*-propylamine)

#### Reaction of PA with NH_2_ (pathways C → F)

The dehydrogenation of PA via amino group (^−^NH_2_) prompts the formation of an ammonia (NH_3_) group, that is an important moiety for proton related reactions.

**Figure 6:**
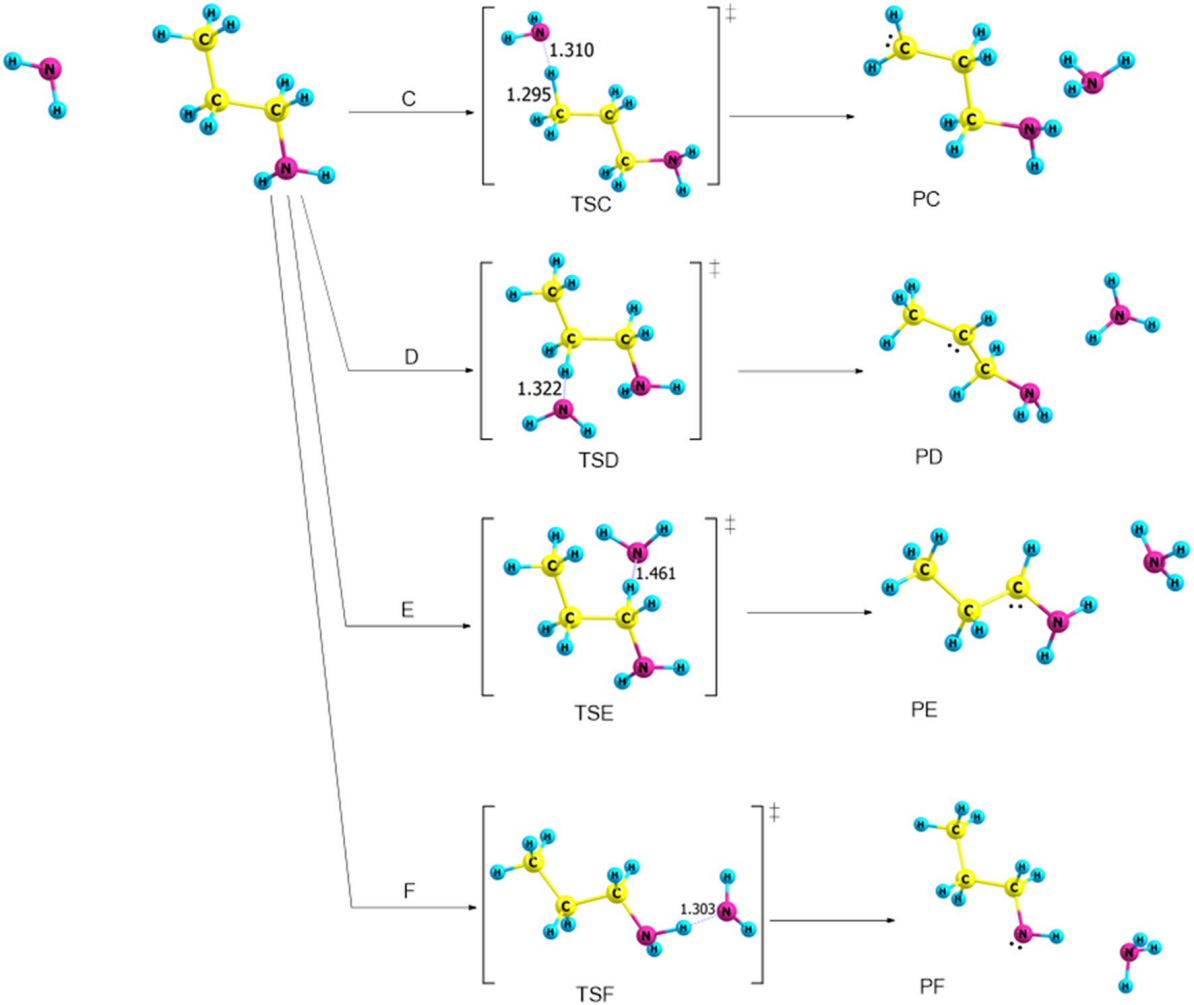
Proposed reaction mechanisms for the dehydrogenation of *n*-PA (pathways **C **→ **F**). Optimized structures at B3LYP/6-311++G(3df,3dp).

**Figure 7:**
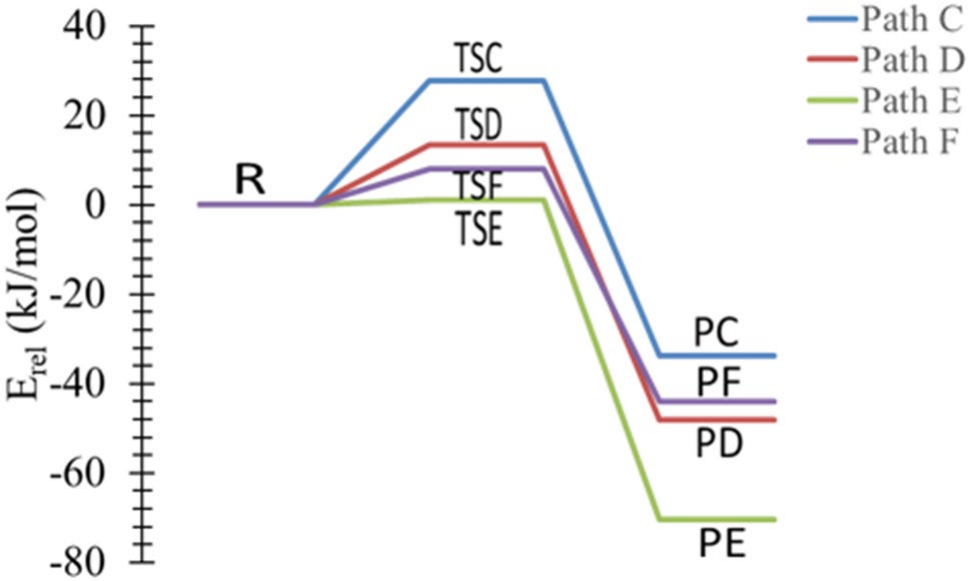
The PED of the protonation reactions of n-PA, pathways **C** → **F**, calculated at B3LYP/6-311G++(3df,3pd).

The NH_2_ group is electron releasing at proton in α-, β-, or γ-carbons, which results in different kinetic, thermodynamic, and bond parameters. Pathway **C** shows the proton transfer from γ-carbon to NH_2_ with an increase of the bond length between γ-carbons and H atom to be 1.295 Å. The distance between N–H decreases to 1.310 Å via TSC (Fig. 6). The energy barriers are calculated to be 34, 27, and 24 kJ mol^-1^ at the B3LYP/6-31G(d), B3LYP/6-311++G(3df,3pd), and CBS-QB3 levels of theory (Table 2), respectively. It is worth noting that for pathway **C**, the PED in Fig. 7 shows that the reaction is thermodynamically reversible with low products energies relative to the transition states.

**Table 2:** Kinetic parameters (E_a_ and ΔG^‡^) for the protonation of *trans*-PA (in kJ mol^−1^) at 298.15 K.

Transition states	B3LYP/6-31G(d)		B3LYP/6-311++G(3df,3pd)		CBS-QB3	
	Ea	ΔG^‡^	Ea	ΔG^‡^	Ea	ΔG^‡^
TSC	34	52	27	55	24	38
TSD	37	48	66	48	13	37
TSE	3	18	1	15	1	13
TSF	11	20	11	32	8	19

In pathway **D**, the proton transfers from β-carbon to NH_2_ and the distance between β-carbons and H atom is 1.322 Å. TSE indicates that the dehydrogenation occurs through abstracting the proton from α-carbon. Likewise, pathway **F** represents the formation of CH_3_CH_2_CH_2_NH and NH_3_ through TSF, with the proton abstracted from amine site, as shown in Fig. 6.

The activation energy of TSF at the B3LYP/6-31G(d) level of theory is in good agreement with B3LYP/6-311G++(3df,3pd) with an energy value of 11 kJ mol^−1^. For the CBS-QB3, the value obtained is 8 kJ mol^−1^. According to Table 2**,** the lowest energy barrier has been calculated for TSE, with a value of 1 kJ mol^−1^ at CBS-QB3. However, pathway **E** is considered to be the most plausible mechanism in dehydrogenation reactions via NH_2_ due to the lower barrier.

#### Reaction of PA with H^+^ (pathways G → J)

**Figure 8:**
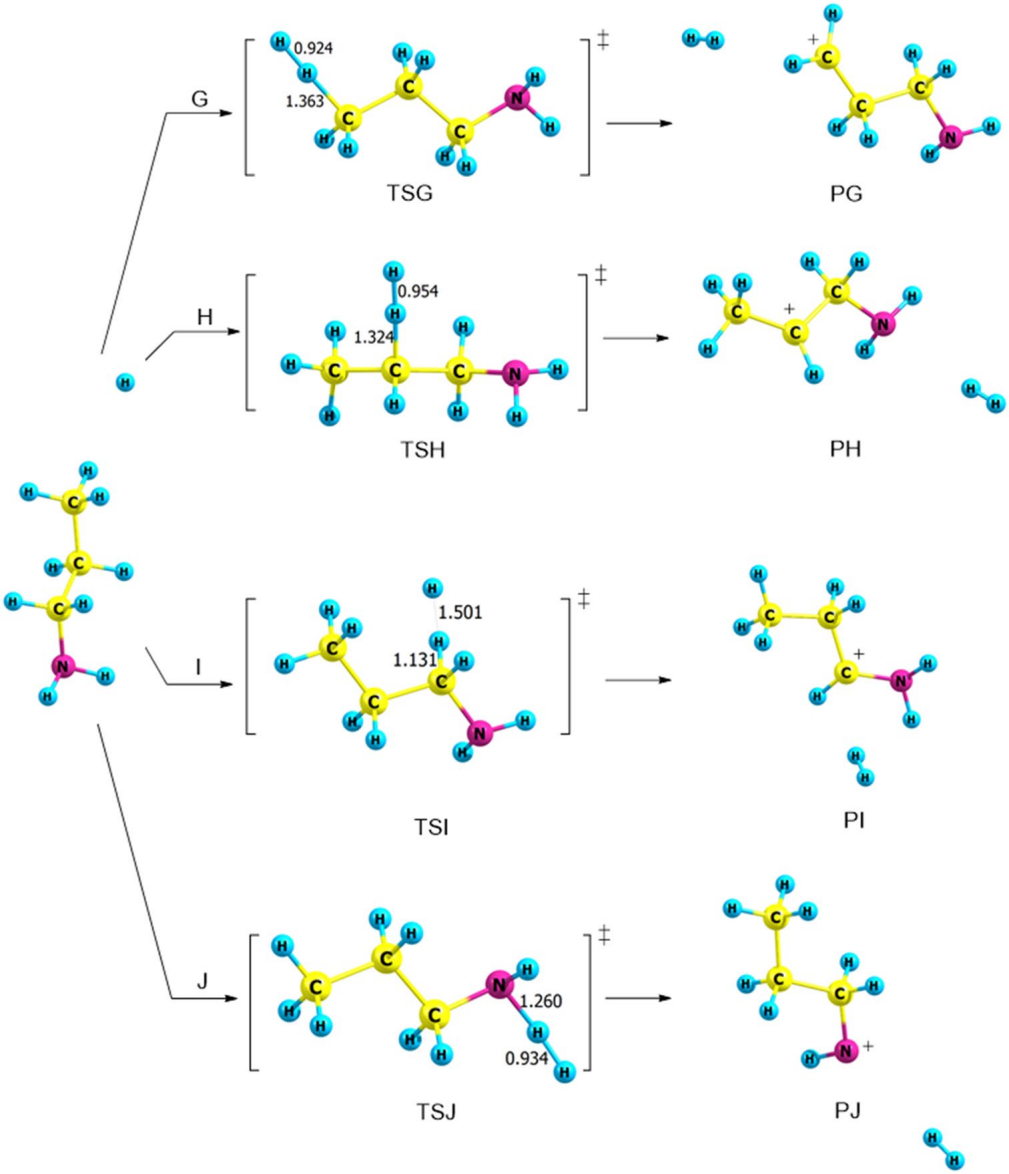
Proposed reaction mechanisms for the dehydrogenation of *n*-PA (pathways **G **→ **J**)**.** Optimized structures at B3LYP/6-311++G(3df,3dp).

The propylamine acts as a week base because the nitrogen atom has a lone pair of electrons that can accept a proton. This section examines the formation of H_2_ and propylamine cation products through four separate pathways, designated as pathways G → J (Fig. 8). In pathway **G**, the hydrogen cation acts as Lewis acid (electron pair acceptor) and abstracts a proton from **γ**-carbon to form H_2_ and **γ**-propylamine cation. With the same mechanism through TSH and TSI, hydrogen is eliminated from α- and β-carbon, and H_2_ and α-and β-propylamine cation can be formed. Likewise, pathway **J** represents the formation of CH_3_CH_2_CH_2_NH^+^ and H_2_ (PJ) through TSJ with hydrogen being abstracted from the amine site, as shown in Fig. 8.

**Figure 9:**
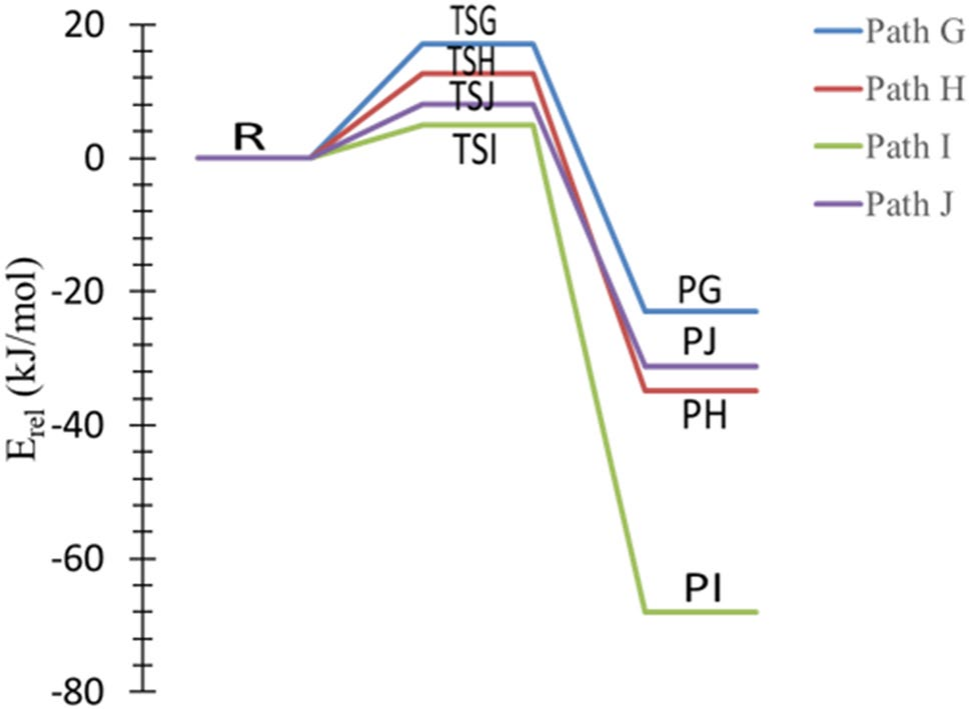
The PED of the protonation reactions of PA, pathways **G** → **J**, calculated at CBS-QB3.

The calculated activation energies are displayed in Table 3 at different levels of theory. The activation energy of TSI is low compared to other pathways with a value of 5 kJ mol^−1^ at the B3LYP/6-311++G(3df,3pd) level of theory and a value of 7 kJ mol^−1^ at the CBS-QB3, see Table 3 and Fig. 9. The thermodynamic parameters indicate that the reaction is exothermic by 71 kJ mol^−1^ and exergonic by 72 kJ mol^−1^ at the B3LYP/6-311++G(3df,3pd).

**Table 3:** Kinetic parameters (E_a_ and ΔG^‡^) for the protonation of *trans*-PA (in kJ mol^−1^) at 298.15 K.

Transition states	B3LYP/6-31G(d)		B3LYP/6-311++G(3df,3pd)		CBS-QB3	
	Ea	ΔG^‡^	Ea	ΔG^‡^	Ea	ΔG^‡^
TSG	21	37	17	38	17	40
TSH	13	30	13	17	18	21
TSI	8	4	5	6	7	8
TSJ	7	14	11	23	8	15

#### Reaction of PA with CH_3_^+^ (pathways K → M)

**Figure 10:**
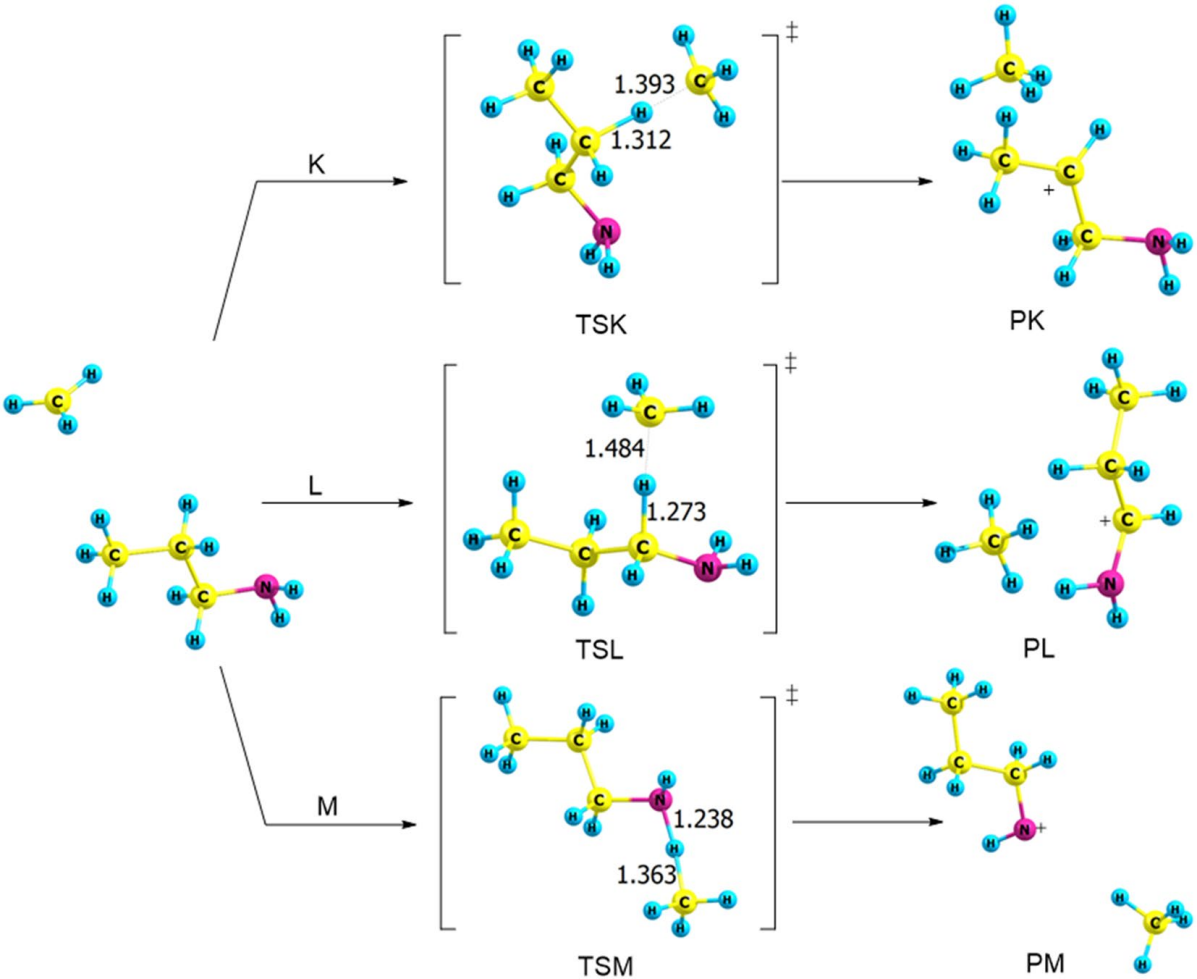
Proposed reaction mechanisms for the dehydrogenation of *n*-PA (pathways **K → M**). Optimized structures at B3LYP/6-311++G(3df,3dp).

Three pathways are studied to understand the reaction mechanisms of dehydrogenation process of PA with methyl cation, denoted as pathways **K**, **L** and **M** as shown in Fig. 10. All transition states are described by a proton removable from different sites in PA; α- and β-carbon in addition to N- atom in the amine group, to form methane and propylamine cation.

**Figure 11:**
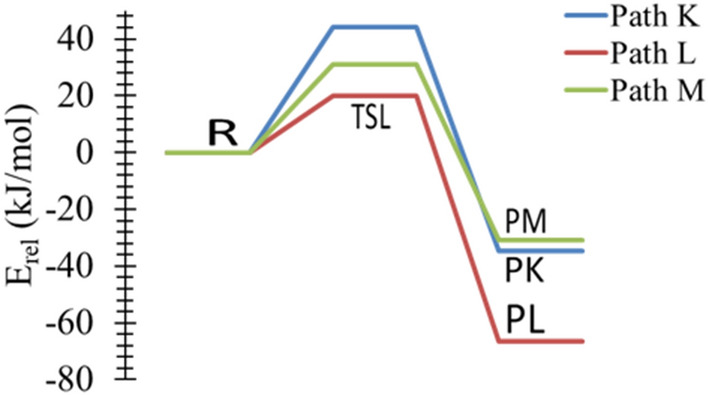
The PED of the protonation reactions of n-PA, Pathways **K → M**, calculated at the CBS-QB3.

Table 4 reports the activation energies and Gibbs energies of activation for pathways **K → M**. The highest activation energy of TSK is 44 kJ mol^−1^ at the CBS-QB3, differing by no more than 2 kJ mol^−1^ from other reported levels of theory, Fig. 11. Moreover, the activation energy of TSK at the B3LYP/6-311G++ (3df,3pd) level of theory is 42 kJ mol^−1^, which is the same as the results of the B3LYP/6-31G(d) level of theory. The most plausible reaction mechanism is pathway **L** via TSL with an activation energy of 20 kJ mol^−1^ at the B3LYP/6-31G(d) level of theory. Moreover, adding polarization and diffuse functions increase the activation energies by 8 kJ mol^−1^. This is comparable with the values calculated at the CBS-QB3, with the barrier of 20 kJ mol^−1^. Thus, based on these results, the reaction is kinetically favored (lower barrier height) and is the most plausible pathway, forming methane and CH_3_CH_2_CHNH_2_ (**PL**).

**Table 4:** Kinetic parameters (E_a_ and ΔG^‡^) for the protonation of *trans*-PA (in kJ mol^−1^) at 298.15 K.

Transition states	B3LYP/6-31G(d)		B3LYP/6-311++G(3df,3pd)		CBS-QB3	
	Ea	ΔG^‡^	Ea	ΔG^‡^	Ea	ΔG^‡^
TSK	42	50	42	65	44	64
TSL	20	43	28	56	20	52
TSM	26	36	34	58	31	41

#### Bimolecular reaction of *n*-PA (pathways N → P)

**Figure 12:**
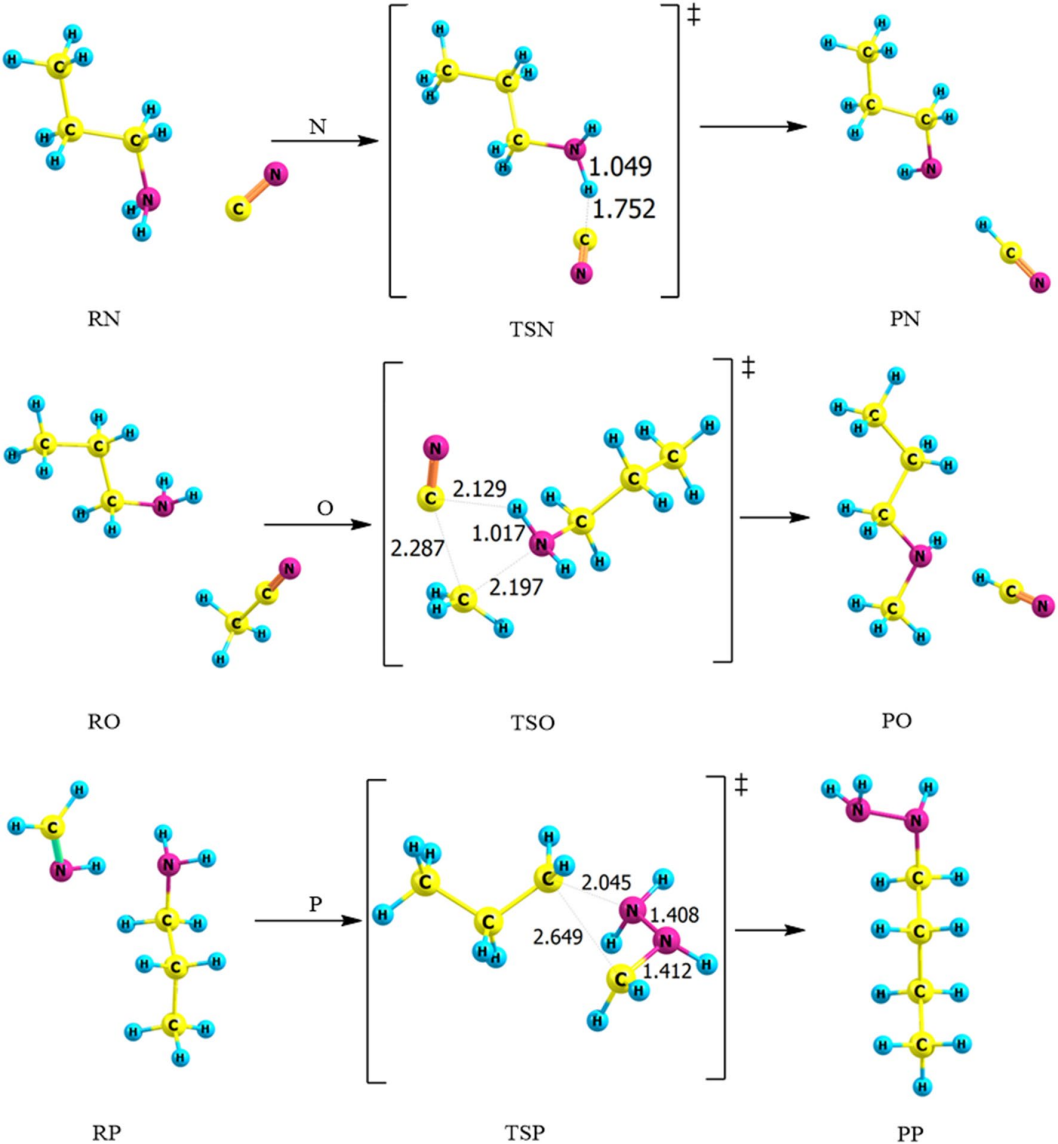
Proposed reaction mechanisms for the bimolecular reactions of *n*-PA (pathways **N **→ **P**). Optimized structures at B3LYP/6-311++G(3df,3dp).

Three primary possible pathways for the bimolecular reaction of n-propylamine with cyanide ion (CN^−^), acetonitrile (HCN) and methanimine (CH_2_NH) are explored and denoted as pathways **N**, **O**, and **P**, respectively. In pathway **N**, hydrogen cyanide is recovered by SN2 reaction of gas-phase PA with cyanide ion via transition state TSN. In Fig. 12, there are some noteworthy changes in the bond lengths and torsion angles. Particularly, the dehydrogenation of PA happens in a concerted step through the separation of the N–H bond. Hydrogen cyanide is essentially created, where the bond is elongated by 0.038 Å to become 1.049 Å, forming CH_3_CH_2_CH_2_NH. For pathway **O**, TSO shows that there is an elongation in bond length of the N–H in propylamine from 1.015 to 1.017 Å. The distance between carbon atom in cyanide atom and H in PA decreased around 0.957 Å. Likewise, butyl hydrazine formed through TSP by the addition of methanimine to PA, the C–N bond in methanimine is elongated by 0.150 Å. On the other hand, the atoms in the C–C bond in PA and methanimine approach each other, and the distance between them is become 2.649 Å.

Table 5 shows that the energy values obtained for the three pathways using the B3LYP/6-311G++(3df,3pd) level of theory are 16, 387, and 447 kJ mol^−1^, respectively. In TSN, the overall activation energy of 21 kJ mol^−1^ at B3LYP/6-31G(d) level of theory is the highest, relative to the other levels of theory. The barrier was found to be 16 kJ mol^−1^ at B3LYP/6-311++G(3df,3pd) which is in excellent agreement with CBS-QB3 value of 17 kJ mol^−1^. In TSO, the activation energy at the CBS-QB3 level of theory with a value of 316 kJ mol^−1^ is lower than the DFT energy values. It merits referencing that utilizing the diffuse and polarization functions on TSN**,** TSO, and TSP with the B3LYP method lead to a decrease in the energy barrier. By comparison, pathway **N** is the most favorable pathway with a value of 16 kJ mol^−1^.

**Table 5:** Kinetic parameters (E_a_ and ΔG^‡^) for the protonation of *trans*-PA (in kJ mol^−1^) at 298.15 K.

Transition states	B3LYP/6-31G(d)		B3LYP/6-311++G(3df,3pd)		CBS-QB3	
	Ea	ΔG^‡^	Ea	ΔG^‡^	Ea	ΔG^‡^
TSN	21	14	16	18	17	20
TSO	384	394	387	401	316	326
TSP	449	464	447	468	448	467

## The thermodynamic parameters of the dehydrogenation reaction of propylamine

The thermodynamic parameters (∆H and ∆G) for the dehydrogenation reaction of PA along with its proposed reactions are studied at all the levels of theory and reported in Table 6. The dehydrogenation reactions of PA are exceptionally exothermic and exergonic at all levels of theory. Nevertheless, the unimolecular dissociation reactions of PA (Pathways **A** and **B**), the bimolecular reactions of the propylamine with acetonitrile (Pathway **O**) and methanimine (Pathway **P**), are endothermic and endergonic, at all levels of theory. In view of the results, we infer that the pathway **E** has the lowest thermodynamic parameters values; therefore, they are more spontaneous and plausible reactions to occur in the atmosphere.

**Table 6:** The thermodynamic parameters of the dissociation reaction of propylamine.

Pathway	B3LYP/6-31G(d)		B3LYP/6-311++G(3df,3pd)		CBS-QB3	
	ΔH	ΔG	ΔH	ΔG	ΔH	ΔG
A	152	138	158	141	157	144
B	116	71	76	85	164	120
C	− 22	− 18	− 38	− 25	− 36	− 24
D	− 20	− 22	− 22	− 20	− 51	− 40
E	− 61	− 55	− 86	− 79	− 73	− 72
F	− 44	− 48	− 44	− 40	− 46	− 51
G	− 16	− 18	− 25	− 34	− 22	− 21
H	− 33	− 40	− 37	− 52	− 34	− 44
I	− 73	− 74	− 71	− 72	− 70	− 70
J	− 44	− 53	− 34	− 41	− 37	− 44
K	− 31	− 43	− 30	− 28	− 37	− 30
L	− 69	− 63	− 69	− 64	− 69	− 64
M	− 45	− 54	− 40	− 46	− 33	− 46
N	− 71	− 80	− 67	− 69	− 70	− 70
O	43	57	51	66	48	60
P	24	42	31	55	32	54

## Conclusions

An elaborate computational study for the gas-phase dehydrogenation reaction of n-propylamine has been performed in detail using quantum-chemical calculations. Two significant pathways for the unimolecular reaction of PA, eleven for the dehydrogenation reactions, and three for the bimolecular reactions with ^−^CN, H_3_C_2_N, H_2_CNH were studied, with a total of 16 pathways. The enhanced geometries including the R's, TS's, I's, and P's were determined. Besides, the potential energy diagram (PED) was described using the DFT and CBS-QB3 methods. For each proposed mechanism, thermodynamic and kinetic parameters were calculated using the DFT and CBS-QB3 methods. Among DFT functionals, the B3LYP/6-31G(d) level of theory is the most used one. However, it still has challenges to predict the accurate activation energies as it misses the dispersion effect and van der Waals interactions. The CBS-QB3 was used due to the accurate description of kinetics and energy barriers. It is worth noting that the B3LYP/6-311++G(3df,3pd) and CBS-QB3 methods produce comparable agreement in terms of energy values within no more than 10 kJ mol^–1^. This indicates that the B3LYP/6-311++G(3df,3pd) performs very well and can be used to study such systems. The IRC calculations were performed to investigate and approve the association of the TS's with the proper minima (I's, R's, and P's) for each proposed pathway. It is worth mentioning that all dissociation reactions mechanism occurs in a concerted step as an exothermic process, except in the case of the unimolecular decomposition and pathways O and P that are considered as endothermic. These findings are important for future research with acidic or alkaline catalysts.
